# A systematic classification of *Plasmodium falciparum *P-loop NTPases: structural and functional correlation

**DOI:** 10.1186/1475-2875-8-69

**Published:** 2009-04-18

**Authors:** Deepti Gangwar, Mridul K Kalita, Dinesh Gupta, Virander S Chauhan, Asif Mohmmed

**Affiliations:** 1 Malaria Group, International Center for Genetic Engineering and Biotechnology, Aruna Asaf Ali Marg, New Delhi-110067, India; 2 Structure & Computational Biology Group, International Center for Genetic Engineering and Biotechnology, Aruna Asaf Ali Marg, New Delhi-110067, India

## Abstract

**Background:**

The P-loop NTPases constitute one of the largest groups of globular protein domains that play highly diverse functional roles in most of the organisms. Even with the availability of nearly 300 different Hidden Markov Models representing the P-loop NTPase superfamily, not many P-loop NTPases are known in *Plasmodium falciparum*. A number of characteristic attributes of the genome have resulted into the lack of knowledge about this functionally diverse, but important class of proteins.

**Method:**

In the study, protein sequences with characteristic motifs of NTPase domain (Walker A and Walker B) are computationally extracted from the *P. falciparum *database. A detailed secondary structure analysis, functional classification, phylogenetic and orthology studies of the NTPase domain of repertoire of 97 *P. falciparum *P-loop NTPases is carried out.

**Results:**

Based upon distinct sequence features and secondary structure profile of the P-loop domain of obtained sequences, a cladistic classification is also conceded: nucleotide kinases and GTPases, ABC and SMC family, SF1/2 helicases, AAA+ and AAA protein families. Attempts are made to identify any ortholog(s) for each of these proteins in other *Plasmodium *sp. as well as its vertebrate host, *Homo sapiens*. A number of *P. falciparum *P-loop NTPases that have no homologue in the host, as well as those annotated as hypothetical proteins and lack any characteristic functional domain are identified.

**Conclusion:**

The study suggests a strong correlation between sequence and secondary structure profile of P-loop domains and functional roles of these proteins and thus provides an opportunity to speculate the role of many hypothetical proteins. The study provides a methodical framework for the characterization of biologically diverse NTPases in the *P. falciparum *genome.

The efforts made in the analysis are first of its kind; and the results augment to explore the functional role of many of these proteins from the parasite that could provide leads to identify novel drug targets against malaria.

## Background

Despite encouraging advances in vaccine development, malaria remains the most serious and widespread parasitic disease of humans. Each year, approximately 300–500 million people become infected with malaria and two to three million die as a result [1]. The availability of the complete genome sequence of *Plasmodium falciparum*, the causative agent of fatal cerebral malaria, has opened new avenues to identify genes important for the parasite's survival. This information can be utilized for the development of effective drugs or vaccines against the parasite. Unfortunately, nearly 60% of the *P. falciparum *genome (5411 proteins) has been designated as hypothetical proteins as they lack sequence similarity to any protein known to date [2]. This large and unexplored group of hypothetical proteins may contain proteins that play an important role in physiological pathways specific to the malaria parasite. The functional interruption of such proteins/pathways without deleterious consequences to the host should be the one of the primary tasks in data mining. A pipeline of systematic studies is thus required to elucidate the functional relevance of such proteins in the parasite's survival.

A number of these hypothetical proteins (unknown protein function) in *P. falciparum *genome contain a P-loop NTPase domain. Briefly, the P-loop NTPases constitute a large super-family of proteins [3] and are involved in disparate physiological processes. For example, processes include translation, transcription, replication and DNA repair, intracellular trafficking, membrane transport and activation of various metabolites [3-5]. The P-loop NTPases carry out such diverse cellular functions by hydrolyzing the α-β phosphate bond of a bound nucleotide triphosphate i.e. ATP or GTP [6].

Based on amino acid sequence, the P-loop NTPase fold is characterized by the presence of a N-terminal Walker A motif, represented by a flexible loop joining a β-strand and an α-helix. The loop typically adopts the sequence pattern GxxGxGK [ST], whose function is to properly position the triphosphate moiety of a bound nucleotide. The distal Walker B motif (hhhhDE) contains a conserved aspartate (less commonly glutamate residue). The motif is situated terminally in a β-strand and binds a water-bridged Mg^2+ ^ion [5,7]. On structural aspects, the P-loop NTPases are α-β proteins that contain regularly recurring α-β units with the five β-strands (β1–β5). The β-strands forms a central core arranged in the order β(5-1-4-3-2) or β(5-1-3-4-2), surrounded by α-helices on both sides [8,9]. The P-loop NTPases can be divided into two groups: one group includes the nucleotide kinases and the GTPases where the β-strand leading to the P-loop and the Walker B strand are direct neighbors. The second group includes AAA, ABC, SF1/2 helicases and RecA/F1 ATPases and is characterized by an additional β-strand inserted between the P-loop strand and the Walker B strand [10-12]. Despite these basic common sequence and structural features, the P-loop NTPases exhibit extreme sequence divergence. The huge sequence diversity had so far hampered a clear understanding of the phylogenetic relationships within these P-loop proteins. The entire complement of the P-loop NTPases of an organism varies considerably in sequence and the functional aspects. Therefore, identification and phylogenetic classification of the P-loop NTPases of an organism may provide insights into distinct physiological processes involving these proteins. Since the malaria parasite resides inside the host cells during most part of its life cycle, some of these processes such as trafficking of proteins and translocation of metabolites might be unique to the parasite and involve P-loop NTPases. In the present study, a comprehensive survey of the P-loop NTPases in *P. falciparum *is carried out by analyzing the sequence and structural features of the NTPase domain of functionally diverse proteins. Further, based on these identified features, a few of these hypothetical proteins are classified. For robustness of the analysis, the traditional cladistic and phylogenetic tree approaches (based on sequence and structural motifs of P-loop NTPases) are also combined. The study underlines the evolutionary information in addition to other sequence-structure features and thus, to develop a systematic classification of the P-loop NTPases in the *P. falciparum*. The study has also facilitated identification of some of the parasite specific P loop-NTPases as putative novel drug targets against the parasite.

## Methods

### Retrieval of sequences with classical Walker A and Walker B motifs

An initial computational search of the complete *P. falciparum *genome for the Superfamily: P-loop NTPase (SSF52540) revealed 302 proteins; 67 out of which are annotated as hypothetical proteins. In addition, to extract the proteins with classical Walker A and Walker B motifs and their variants, the motif search tool of PlasmoDB release 4.4  is used. These two motifs are represented by GxxGxGK [TS] and hhhhDE patterns, respectively where 'x' is any of the 20 amino acids and 'h' is any hydrophobic residue. It led to the inclusion of those proteins which have been annotated as P-loop NTPases by PlasmoDB and Pfam databases. From this analysis, an initial set of 120 P-loop NTPase sequences containing both Walker motifs is obtained. Further, for each of these proteins; the sequence regions corresponding to the P-loop domain only (comprising both Walker regions) are extracted. Variation in the sequence length (171 to 8094 amino acids) and Walker A and Walker B motifs is observed for most of these proteins. The unavailability of crystal structures of most of these proteins is a major hindrance in the structure based multiple alignments and their systematic analysis; hence only 97 proteins in which the P-loop domain encompasses a length of 300 to 400 residues are studied, to facilitate in sequence based multiple alignments (Additional file 1). The refined dataset is then used for secondary structure and phylogenetic analyses, followed by classification and orthology studies. Apart from above analysis, the protein sequences are also screened using signalP 3.0, TMHMM v2.0 and TMpred tools to predict signal and transmembrane domains. The absolute expression profiles of these proteins that are temperature synchronized are also obtained from PlasmoDB.

### Functional classification using Pfam v21.0

Since the P-loop NTPases are known to belong to functionally diverse groups therefore a domain analysis of dataset of 97 *P. falciparum *P-loop proteins is performed for functional classification of these proteins using Pfam v21.0 database of domains. The proteins are functionally correlated based on the domains present with E-values with a threshold of 10^-2^.

### Secondary structure analysis of proteins

To perform secondary structure analysis of the P-loop domain of the dataset of 97 proteins, a representative dataset of 533 seed protein sequences is generated pertaining to Pfam accession ID: PF00004, PF07724, PF07726, PF04326 and PF07728. The secondary structure is predicted by the standalone version of PSIPRED v2.0, which is a simple and reliable method, incorporating two feed-forward neural networks that perform an analysis on output obtained from the initial run of PSI-BLAST at the cut-off E-value of 0.001 (Blast v2.2.4) [13]. PSIPRED predicts the secondary structure for each residue and provides a confidence score for three types of secondary structures: helix, sheet and coil. The sequences are aligned according to the secondary structure for AAA+ proteins to classify them into classical AAA or AAA+ and other super-families based on the structural conformations and domain organizations. Multiple alignments are obtained using CLUSTALW (1.83) [14-16], which are manually refined to reflect the available structural information.

### Phylogenetic analysis

The phylogenetic relationships amongst various classes of proteins are determined using the Phylip 3.67 package [17]. PROTDIST is used on the 97 sequences to calculate a distance matrix according to the Dayhoff PAM probability model [18]. The computed distances represent the expected fraction of amino acid substitutions between each pair of sequences. The distance matrix is then used to estimate phylogenies using the neighbour joining (NJ) method. Bootstrapping is carried out using SEQBOOT (1,000 replicates for the PAM model of substitution). CONSENSE is used to compute the consensus tree by the majority rule method. The final unrooted tree diagram is generated using TreeView .

### Identification of orthologous protein sequences using OrthoMCL

To identify the possible drug targets in terms of orthology of proteins between *P. falciparum *and its human host and with other Apicomplexans, OrthoMCL algorithm is used [19], which performs the Markov Clustering (MCL) to group orthologs and paralogs across proteins of multiple organisms. The stand-alone version 1.3 of OrthoMCL is obtained from . Protein FASTA files of each of the genome is given as input to the algorithm. An all-against-all BLASTP analysis (at E value of 10^-5^) is carried out using OrthoMCL. The blast output, which describes genes paired by BLAST matches, the E-value, and the identity percentage and the related HSP information, is parsed to OrthoMCL. The evolutionary related proteins are interlinked in a similarity graph matrix. MCL  is then invoked to split mega-clusters as an analogous process of manual review in COG construction (Clusters of Orthologous Groups). As a result, different clusters of orthologous proteins are created.

## Results and discussion

### Sequences with P-loop motifs: Generation of dataset

Using consensus sequences of Walker A (GxxGxGK [ST]), Walker B (hhhhDE) motifs and their variants along with other proteins annotated as P-loop NTPases (PlasmoDB), a dataset of 97 *P. falciparum *protein sequences is generated. These proteins showed variations in the length of the P-loop NTPase domains having insertions and repeat regions between Walker A and Walker B motifs. In addition, some of the proteins showed variations in the P-loop motifs compared to canonical forms, suggesting their divergence from other P-loop NTPases. These sequences are further analysed for protein domain organization, secondary structure and phylogenetic and orthologous relationships. A list of all the 97 proteins and their characteristics is provided in Additional file 2.

### Functional classification based on domains and secondary structural analysis

**Figure 1:**
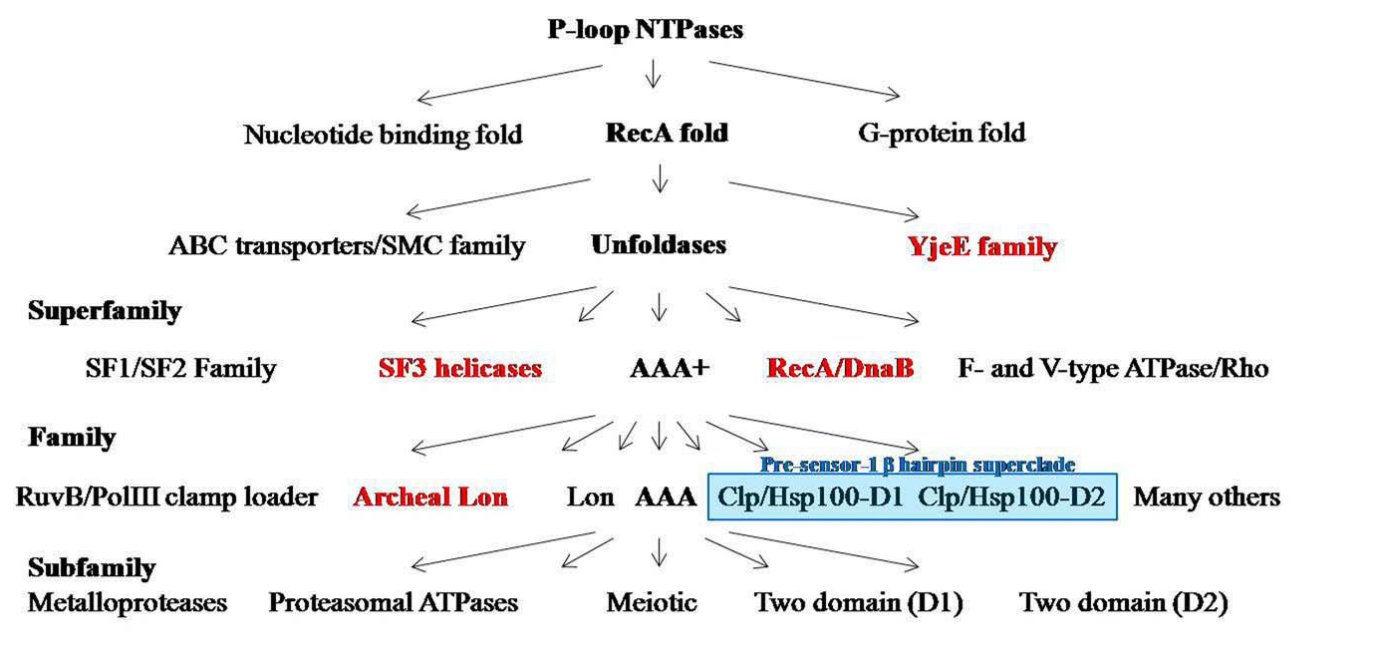
**Schematic representation of classification of P-loop NTPases into families, super families and clades (following Leipe et al., 2002; 2003)**. The families marked in red are not observed in *Plasmodium falciparum*. The clades that together form the Pre-sensor-1 β hairpin superclade are highlighted in blue.

Analysis of the secondary structures of the P-loop NTPase domain and presence of the Pfam domains served as the basis for functional classification of these proteins. Typically, the functional groups have been defined based upon the presence of Pfam domains and the hierarchical classification of individual proteins is carried out on the basis of structural analysis of P-loop domains in these proteins using PSIPRED (Figure 1) [20,21]. Based upon these studies; the repertoire of *P. falciparum *P-loop NTPases is classified into clades, families and subfamilies (Additional file 2).

#### Kinase GTPases

In this class two myosin binding proteins of molecular motors, PFL1435c (myosin d) and PF13_0233 (myosin a) are identified. Unlike other myosins, PFL1435c contains long asparagine repeats between the Walker A and Walker B motifs. The orthologs of PF13_0233 protein are also found to be present in other *Plasmodium *species as well as in a closely related apicomplexan organism (*Toxoplasma gondii*); suggesting its evolutionary conservation. Four other kinases are also identified (MAL13P1.148, PF11_0416, PFE0175c and PFF0675c), that showed major variation in the P-loop domain organization; the P-loop domain of these proteins lack a classical Walker B motif. Although a total of 99 protein kinases have been identified in *P. falciparum *genome [22], only PF13_0334 is found to contain the classical P-loop NTPase fold.

The *P. falciparum *GTPases that have been classified within P-loop NTPases include PF11_0183, a nuclear binding protein and two transcription initiation and elongation factors, PFA0595c and PF13_0069. Based upon Pfam domain analysis along with their secondary structures, the two hypothetical proteins (PFF0810c and PF14_0052) are also classified as Kinase GTPases.

### ASCE/RecA fold division

#### ABC transporters and SMC family

**Figure 2:**
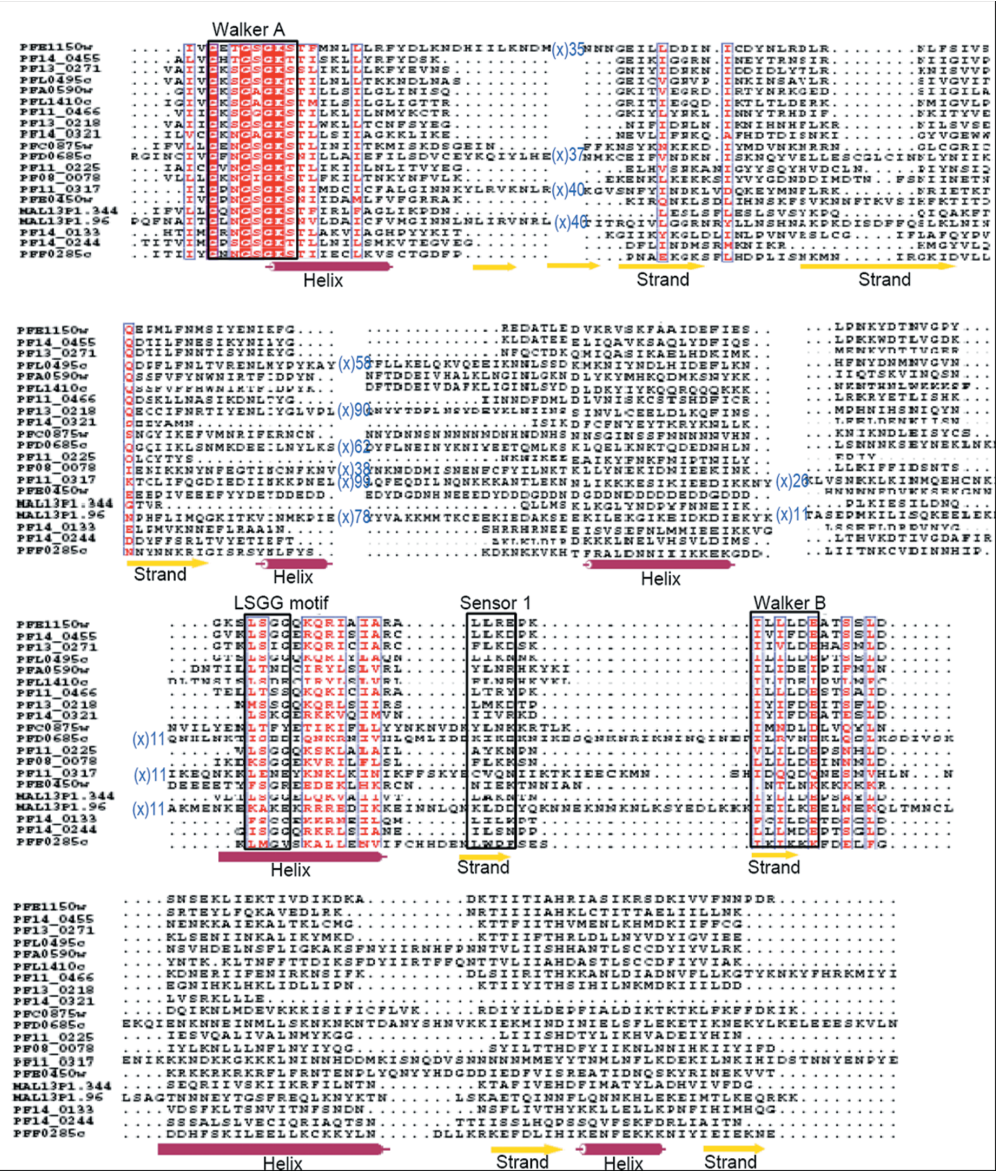
**Multiple sequence alignment of P-loop NTPase domain of *P. falciparum *ABC transporters and SMC proteins**. Conserved residues are in solid red and conserved motifs are boxed in black. The corresponding secondary structure is shown below the alignment. Purple cylinder represents helices and yellow arrows represent β-strands.

ABC transporters are part of a large ATP binding protein family that plays physiological function of translocation of metabolites across the membranes. In *P. falciparum*, 15 ABC transporters are identified that contain core β-strands and helices along with sensor-1 residue, which intercalates between the P-loop and aspartate residue of the Walker B motif. The characteristic signature 'LSGG' of ABC proteins, located upstream of the Walker B motif is found in only five *P. falciparum *ATPases while a hydrophobic residue substitution primarily at the first position of the motif is noticed in a majority of remaining proteins (Figure 2). Based upon these sequence features and secondary structure analysis these 15 ABC transporters are phylogeneticaly assigned into subfamilies following [23]. Out of these 15 ABC proteins, three are full transporter [with two transmembrane domain (TMD) and two nucleotide binding domain (NBD)] PFC0875w, PFE1150w and PF14_0455; and eight are identified as half transporters (with one TMD and one NBD) PF13_0218, PF13_0271, PFL0495c, PFA0590w, PF11_0466, PFL1410c, PF14_0133 and PF14_0244; whereas four other ABC proteins are found to lack the transmembrane domain. Based on these observations, PF13_0218, PF13_0271, PFL0495c, PF14_0455 and PFE1150w have been assigned to the ABCB subfamily, which is known to include multidrug resistant proteins (MDR/TAP). These P-glycoprotein (PgP) homologues of the *P. falciparum *are found to be similar to the mammalian MDR proteins and have been implicated in providing parasite resistance against anti-metabolites [24]. ABC transporters have also been implicated to export proteins (responsible for host modification, virulence and immune responses) in the host via alternative pathways [25]. The PF11_0466 protein belonging to ABCB subfamily, is an ABC transporter, expressed in late trophozoite stages [26] and contains a hydrophobic N-terminal signal sequence. The N-terminal signal sequence is known to be responsible for default trafficking of a parasite protein through endoplasmic reticulum-Golgi apparatus pathway to the parasitophorous vacuole in absence of any organelle targeting signals. It is not clear if PF11_0466 protein is localized to mitochondria or apicoplast; in absence of targeting to any of these organelles it may localize in the plasma membrane or parasitophorous vacuolar membrane and might have novel function(s) in protein trafficking. Two other *P. falciparum *ABC tarns porters, PFA0590w and PFL1410c are suggested to be involved in redox metabolism and might have functional activities of MRP (MDR associated protein) and/or GSSG pumps [27]. These characteristics suggested that these proteins may be classified under the ABCC subfamily. The mammalian MRPs belonging to the ABCC family are associated with extrusion of oxidized glutathione (GSSG) generated during glutathione synthesis, a major element of antioxidant defense for the parasite [28]. Interestingly, the protein PFL1410c showed no homology with any of the known human MRPs and hence can be studied as a potential drug target. PF14_0244 is assigned to the ABCG subfamily a reverse transporter with N-terminal NBD and C-terminal TMD. Further, the proteins without transmembrane region have been classified under the ABCF subfamily; members of this family are not known to be involved in any membrane transport functions [23].

**Figure 3:**
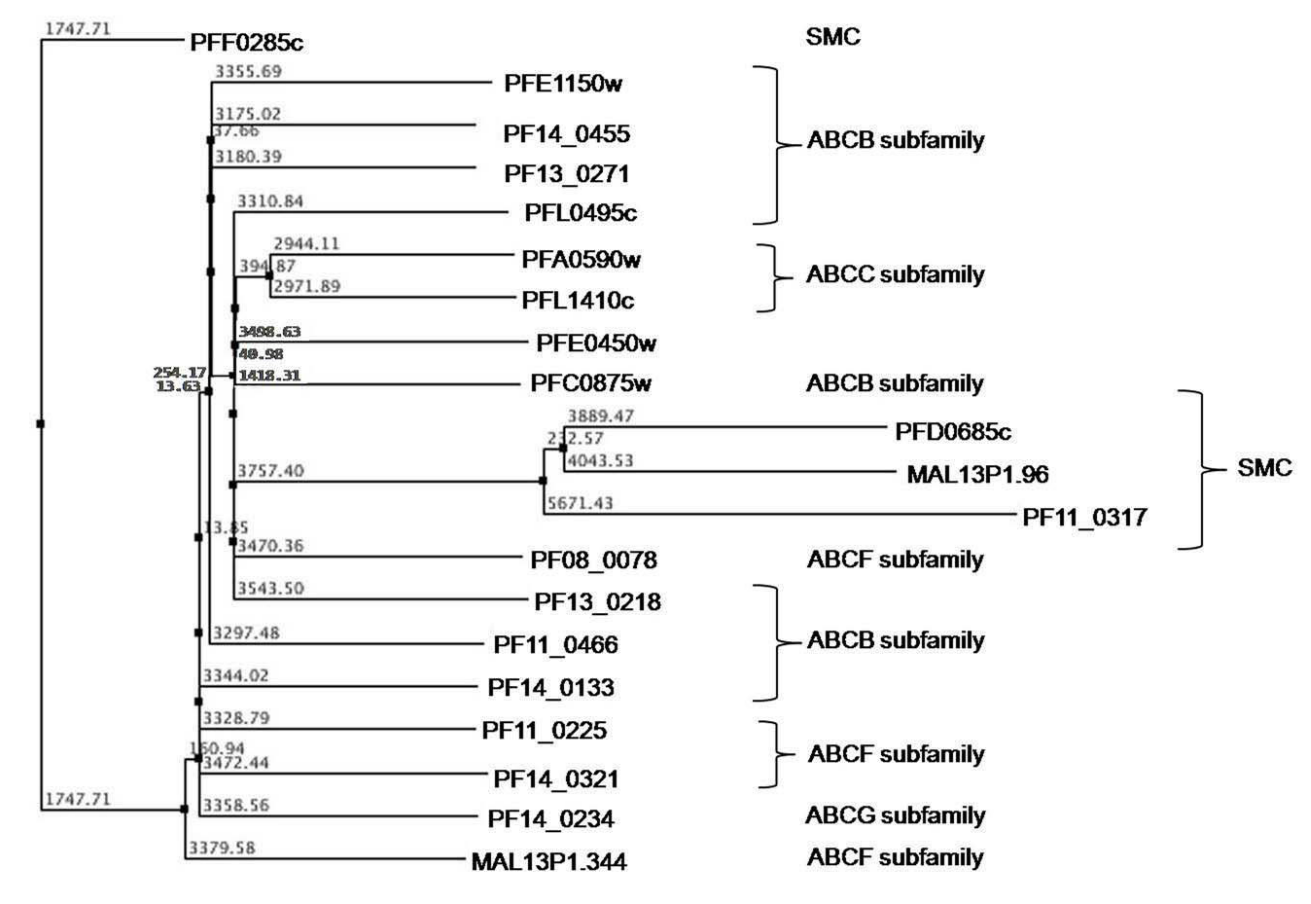
**Phylogenetic classification of *P. falciparum *ABC transporters and SMC proteins based upon sequence and secondary structure homology among their P-loop NTPase domain**. The branch numbers represents the distance values in NJ tree of protein sequences.

Along with the ABC transporters, SMC (Structural Maintenance of Chromosomes) proteins also represent a large family of ATPases involved in chromosome organization like the sister-chromatid cohesion and DNA repair [27]. Apart from high sequence similarity, SMCs also share structural similarity with ABC proteins (Figure 3). Five *P. falciparum *proteins are annotated as putative SMC proteins (PlasmoDB). The *P. falciparum *SMC proteins are found to be highly conserved amongst *Plasmodium *species which suggests that they may play an important role as a component of chromosomal maintenance complex and are probably indispensable for the parasite. Although ABC transporter proteins are functionally different from the SMC proteins, Figure 2 clearly illustrates that the N-terminal region of proteins belonging to these two groups is significantly conserved near the Walker-A motif, extended by two small conserved regions having charged residues (Lys, Arg, Glu) flanked by hydrophobic (Leu, Ile, Ala) or polar residues (Ser, Asn, Gln). Based on secondary structure analysis, three SMC proteins (PFD0685c, MAL13P1.96 and PF11_0317) clustered together as compared to PFF0285c; probably due to two long stretches of continuous helical regions in the PFF0285c protein. Evolutionary studies that the ATP binding domain of proteins belonging to the same ABC subfamily are more closely related to each other than to those belonging to other subfamilies, suggesting their concerted evolution whereas SMC proteins clustered together away from ABC families and have evolved further from these subfamilies (Figure 3).

### Unfoldases

**Figure 4:**
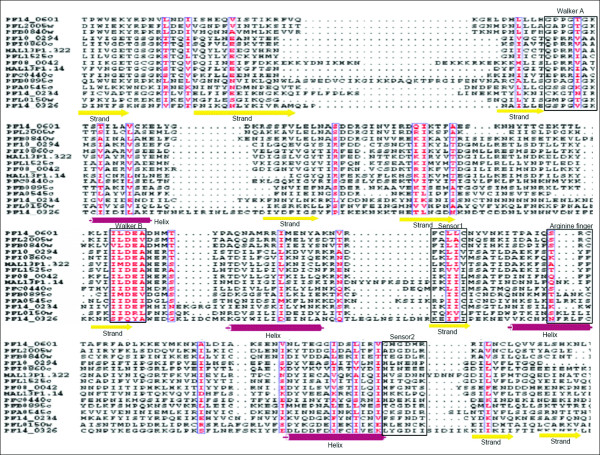
**Multiple sequence alignment of P-loop NTPase domain of *P. falciparum *SF1/SF2 Helicases and Replication factor C (RFC) proteins**. Conserved residues are in solid red and conserved motifs are boxed in black. The corresponding secondary structure is shown below the alignment. Purple cylinder represents helices and yellow arrows represent β-strands.

The unfoldase division of ASCE/RecA fold proteins is characterized by the core P-loop containing Walker A and B motifs, and the presence of arginine finger at the N-terminus of β5 (Figure 4). The division includes SF1/SF2 helicases, F- and V- type ATPases/Rho and AAA+ superfamily of proteins.

#### SF1/SF2 Helicases

Helicases are enzymes that unwind duplex DNA or RNA coupled to nucleoside 5' triphosphate (NTP) binding and hydrolysis [29]. Structural and sequence comparison of these proteins from different organisms have identified seven to nine short, conserved motifs called helicase signature motifs [30]. Most of the known 3'-5' DNA helicases are members of SF1 or SF2. These two superfamilies have similar sets of conserved motifs that are responsible for coupling of ATP hydrolysis to DNA translocation and unwinding. However, sequence homology across SF1 and SF2 families is very weak and limited to the signature sequences, required for NTP binding (Walker A and B motifs) [31]. ATP hydrolysis by SF1 helicases is stimulated only by ssDNA, whereas the ATPase activity of SF2 helicases is stimulated by both ssDNA and dsDNA [32]. In the present study, seven P-loop NTPases in the *P. falciparum *genome are found to contain helicase, HA2 and DEAD/DEAH domain(s), characteristic of DNA/RNA helicases. The secondary structures of these helicases are dominated by helices towards the N-terminal, followed by regions having alternate α-helices and β-sheets. Multiple sequence alignments revealed that although the DEAH box helicases had high sequence conservation throughout the P-loop domain, PF08_0042 and PFC0440c had an 'I' → 'A' replacement in the DEAH box (Figure 4). In addition to some hydrophobic residue conservations among sequences, a sequence pattern 'I [LI]DE [AVIL] is found to be highly conserved. Downstream to the observed pattern, a Ser/Thr residue is found to be well-conserved amongst all the *P. falciparum *helicases.

### AAA+ superfamily

**Figure 5:**
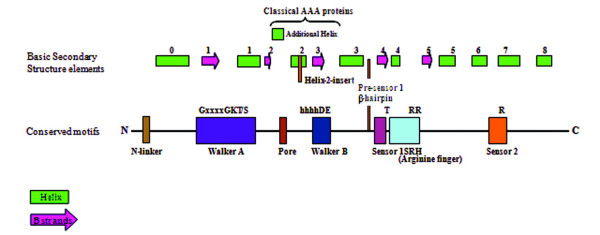
**A schematic showing the organization of secondary structure and key elements of AAA+**. Helical insertions and other variations observed in the domain organization are depicted. The location of characteristic residues within the motifs is marked.

The proteins belonging to AAA+ superfamily are highly diverse and are involved in varied cellular functions [33], but have a common AAA (ATPases Associated with diverse cellular Activities) structural fold [9]. The AAA+ domain contains a characteristic region of high sequence conservation called as the second region of homology (SRH) that distinguishes AAA+ proteins from other P-loop NTPases. The SRH includes a polar residue at its N-terminal known as 'sensor 1' and an 'arginine finger' at the C-terminal helical domain that carries a conserved arginine residue known as 'sensor 2' [34] (Figure 5). The AAA+ superfamily include classical AAA proteins as well as proteins having divergent sequences but classical AAA structural fold. The AAA+ superfamily has been divided into several different clades. The *P. falciparum *AAA+ proteins have been grouped based upon their sequence and structural features and discussed according to their respective functional clades.

#### Clamp loader/RFC clade

**Figure 6:**
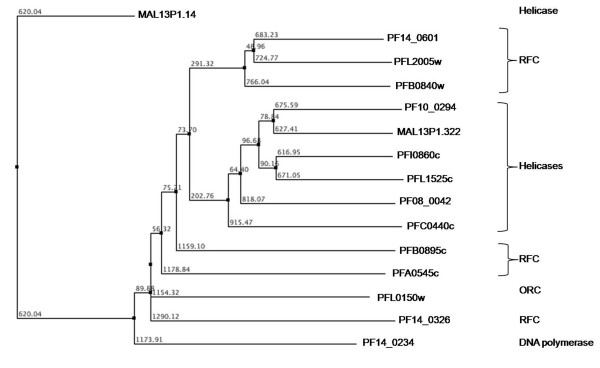
**Phylogenetic classification of *P. falciparum *SF1/SF2 Helicases and Replication factor C (RFC) proteins based upon sequence and secondary structure homology among their P-loop NTPase domain**. The branch numbers represents the distance values in NJ tree of protein sequences.

The clamp loader ATPases have a synapomorphic 'SRC' signature associated with the arginine finger [35]. In the preset study, five *P. falciparum *RFC (replication factor C) proteins are identified following the Walker A and B consensus analysis. Three of these proteins (PF14_0601, PFL2005w and PFB0840w) share high sequence similarities as compared to other two (PFA0545c and PFB0895c). Further, the sequence analysis revealed that the sensor-2 is conserved among all the five RFC proteins while the SRH is conserved in only three proteins (PF14_0601, PFL2005w and PFB0840w). The structural analysis revealed one hypothetical protein (PF14_0326) having secondary structure similar to that of known RFC proteins. This protein showed a single residue substitution in the Walker B motif; 'W' residue replacing A in the {AVIL} pattern (Figure 4). In order to analyse the correlation between the secondary structure of the P-loop domain and functional role of a protein, the helicases and the RFC proteins are studied together. The sequence pattern 'I [LI]DE [AVIL], that is observed for helicases, is also found to be conserved in the RFC proteins. The evolutionary analysis of the helicases along with the replication factor C (RFC) proteins revealed that helicases proteins have a tendency to cluster. The only exception is MAL13P1.14 protein, which falls out of the cluster probably due to extended sequence length in the N-terminal of its P-loop NTPase domain. Three RFC proteins are observed to group closer with the helicases as compared to the other RFC proteins (Figure 6). These results indicate that helicases and RFC proteins might have undergone a convergent evolution since majority of the helicases are found to be similar both at the sequence as well as in the structural organization of the P-loop domain with the RFC proteins.

#### DnaA/CDC6/ORC clade

This clade is defined by the presence of two α-helices after strand 2 (of approximately equal size) that are packed against each other [35]. The only representative of this family is PFL0150w. The N-terminus of the protein had rich low complexity regions and the ATPase domain had secondary structure similar to that of other AAA+ proteins (Figure 6).

#### ClpA/B ATPase clade

**Figure 7:**
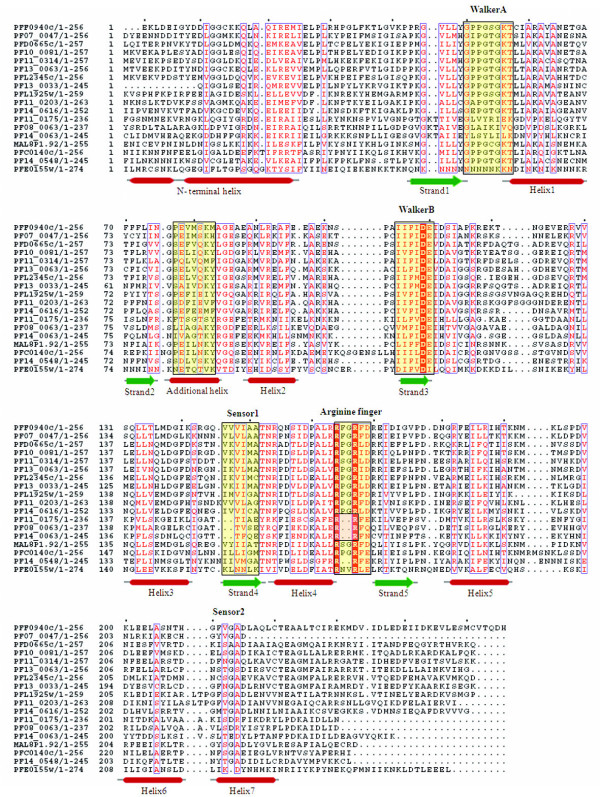
**Multiple sequence alignment of P-loop NTPase domain of *P. falciparum *classical AAA and ClpA/B proteins**. Conserved residues are in solid red and characteristic motifs are boxed in black and shaded in yellow. The corresponding secondary structure is shown below the alignment. Red cylinder represents helices and green arrows represent β-strands. Note the presence of additional helix between strand 2 and helix 2 in these proteins. Also note the lack of conserved glycine in Clp proteins, upstream of arginine finger (boxed in arginine finger).

**Figure 8:**
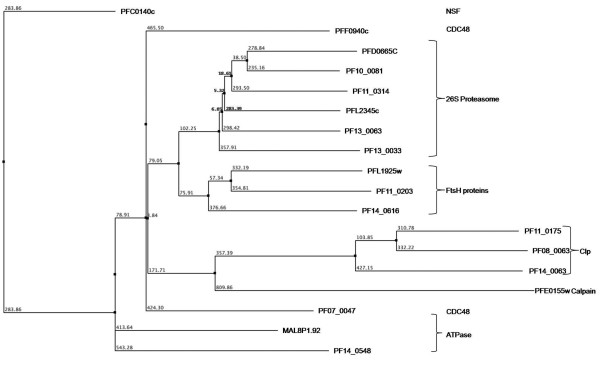
**Phylogenetic classification of *P. falciparum *AAA and ClpA/B proteins based upon sequence and secondary structure homology among their P-loop domain**. The branch numbers represents the distance values in NJ tree of protein sequences.

Members of the ClpA/B ATPases family contain two AAA+ domains that vary from each other in terms of sequence and phylogenetic affinities. The ClpB ATPases contain a middle domain (M domain) between the two ATPase domains as compared to ClpA. The ClpB ATPases play role of important chaperones whereas ClpA ATPases interact with Clp proteases to carry out regulated protein degradation [36]. Two *P. falciparum *proteins (PF14_0063 and PF11_0175) have been annotated as ClpA/B ATPases by PlasmoDB. However, PF14_0063 is found to contain substitution in some of the functionally conserved residues including R → E substitution in the arginine finger and R → A substitution in the sensor 2 region which might render it non-functional. PF08_0063 is identified as another ClpA/B (annotated as hypothetical protein in the PlasmoDB) that also contains the ClpN domain and showed secondary structural organization and domain pattern as other ClpA/B proteins (Figure 7). Evolutionary studies nested these plasmodial ClpA/B homologs within the ClpA/B proteases of bacterial lineage suggesting that they have been acquired from the prokaryotic precursors (Additional file 3). The AAA domain of the *P. falciparum *ClpA/B proteins shared the characteristic features of the classical AAA clade in having the additional small helix downstream of strand 2 except the conserved glycine at the N-terminal of arginine finger. The secondary structure of AAA module of parasite ClpA/B homologs clustered them separately from rest of the AAA proteins (Figure 8). Importantly, the absence of any human ClpA/B homologue renders the *Plasmodium *ClpA/B ATPases as important drug targets.

#### "Pre-sensor-1 β hairpin" (PS1BH) superclade

The remaining lineages of AAA+ namely HslU/ClpX/Lon, MCM, dynein/midasin and other relatives have been unified into a one large monophyletic group. The entire superclade is defined by the presence of an insert between the sensor-1 strand and the preceding helix known as "pre-sensor-1 β hairpin" [35]. In this superclade, two distinct clades are identified, which are further divided into three protein families in the parasite genome.

#### HslU/ClpX/lon clade

The HslU/ClpX clade proteins contain one ATPase domain as compared to ClpA (with two ATPase domains). The ATPase domain of HslU is interrupted by an 'I' domain involved in the substrate binding [37,38]. This clade is supported by an extended loop between strand-2 and helix-2. The HslU (ClpY) and ClpX ATPase interact with the protease partner HslV (ClpQ) and ClpA respectively to form multimeric protease degradation complex machineries as in case of ClpAP. Although ClpX ortholog is absent in *P. falciparum*, the ortholog of HslU (PFI0355c) annotated as ATP-dependent heat shock protein is identified. HslU orthologs are known in apicomplexans and kinetoplastids are predicted to be localized in the mitochondria or plastid [39]. Sequence and structural analysis of PfHsIU confirmed the presence of three characteristic domains of HslU proteins, N-terminal domain (N-domain), Intermediate domain (I domain) and C-terminal domain (C-domain) [40]. The HslU ortholog is an ATP binding regulatory subunit of prokaryotic proteasome complex with HslV/ClpQ threonine proteases. The ortholog of the prokaryotic HslV protease in the malaria parasite has been recently identified and is shown to be functionally important in the parasite [41]. Both HslU and HslV lack any homolog in the vertebrate host and may be considered as a promising drug targets against the parasite. The Lon proteins from archea and bacteria define another family (LON family) within this clade. The PF14_0147 protein is identified as a *P. falciparum *Lon protease that contains the characteristic LAN domain and a Lon-protease domain flanking the AAA domain at the N- and C-termini, respectively.

#### Helix-2 insert clade

As the name suggests, the defining feature of the clade is an insert in helix-2 that folds into two β-strands [35]. In the *P. falciparum *genome, the helix-2 insert clade is represented by Mini Chromosomal Maintenance proteins (MCMs) and dyneins, whereas other members of the clade such as NtrC, YifB and MoxR are not found in the parasite genome.

### MCM family

**Figure 9:**
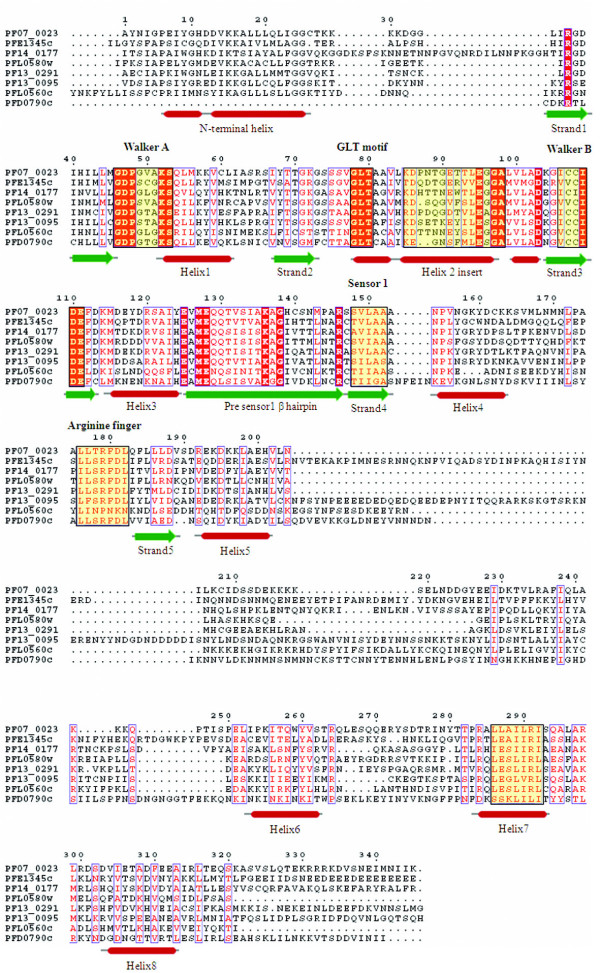
**Multiple sequence alignment of P-loop NTPase domain of *P. falciparum *Mini chromosomal maintenance (MCM) proteins**. Conserved residues are in solid red and characteristic motifs are boxed in black and shaded in yellow. The corresponding secondary structure is shown below the alignment. Red cylinder represents helices and green arrows represent β-strands. Note the presence of helix 2 insert and Pre-sensor 1 hairpin characteristic of this family.

The MCM proteins are essential DNA replication initiation factors and the best characterized among them is a family of six structurally related proteins (MCM2–7) [42]. In the *P. falciparum *database three proteins (PF14_0177, PFD0790c and PFL0560c) have been annotated as MCMs belonging to the MCM2/3/5 family (PlasmoDB). Five other members of the MCM family (PF07_0023, PF13_0095, PF13_0291, PFE1345c and PFL0580w) that have certain minor deviations from the canonical form of P-loop motifs like Gxx [G/x]x [G/A]KS sequence of the Walker A motif are observed. The secondary structure analysis of the AAA region of all these MCM proteins revealed the classical helices and strand pattern. An invariably conserved sequence motif 'GLT' (for the observed pattern of ' [AG] [FL]T' in other organisms) in helix-2 region before the beta-alpha-beta insert is observed. The beta insert in the helix-2 of MCM family members of the malaria parasite is predominantly rich in hydrophobic residues except highly conserved charged residues of ' [KR]D' and 'LE', 5th and 14th positions, respectively, downstream of the GLT motif (Figure 9).

### Dynein family

Dyneins are large molecular motor that transport various cellular cargo by "walking" along cytoskeletal microtubules towards the minus-end of the microtubule, which is usually oriented towards the cell center. Thus, they are called "minus-end directed motors". They contain six tandem AAA+ domains in the same polypeptide chain [9,43]. The parasite genome contains seven representatives (average length of ~ 6000 amino acids) from this family out of which only four proteins (PF14_0626, MAL7P1.162, PF10_0224 and PF11_0240) are annotated at PlasmoDB. Three other proteins PFI0260c, PFL0115w and MAL7P1.89 annotated as hypothetical proteins at PlasmoDB, are identified as dyneins based upon the protein domain analysis. These proteins have two dynein heavy chain domains at their N-terminal named as DHC N-1 and DHC N-2 except in MAL7P1.89 and PF11_0240, which only have the DHC N-2 domain. The PFI0260c protein is also found to contain spectrin-like repeats.

### AAA clade

Proteins belonging to the classical AAA clade contain highly conserved P-loop NTPase domain including SRH. The characteristic feature of the clade is the presence of an additional short helix immediately downstream of strand 2 in the P-loop domain [35]. The AAA clade consists of all ATPases that originally are defined as the members of the AAA superfamily [44]. The *P. falciparum *AAA proteins have been classified under following categories: metalloproteases, proteasomal subunits and 'D1 and D2' proteins. A conserved glycine residue at the N-terminal of the arginine finger is observed in all the members of this clade (Figure 7). Three *P. falciparum *proteins are identified as metalloproteases (pan-bacterial protein family) belonging to the M41 family of peptidases and proteases [45,46]. PFL1925w has been annotated as cell-division protein FtsH whereas PF11_0203 and PF14_0616 have been annotated as hypothetical proteins (PlasmoDB). The domain analysis of these proteins showed that these hypothetical proteins contain a single AAA (Pfam domain id-PF00004) domain fused to the metalloprotease domain (Pfam domain id-PF01434) as in case of other FtsH proteins. Moreover, in all the three metalloproteases, a functional motif 'abXHEbbHbc' where 'a' is most often alanine or serine (instead of valine or threonine residues observed in metalloproteases of other organisms), 'b' is an uncharged residue (tyrosine or alanine here) and 'c' is a hydrophobic residue (leucine or isoleucine here) is observed. Thus both, domain and sequence-structure analysis, suggested that the two hypothetical proteins with characteristic features of the family may be functionally equivalent to FtsH metalloprotease. Upon performing the phylogenetic analysis based on their secondary structures, these three proteins tend to cluster together.

The 26S proteasomes complex is a component of the regulated protein degradation machinery in eukaryotic cell. The 19S regulatory component of the 26S proteasome complex consists of six distinct but closely related proteins (Rpt1–6) [47,48]. These proteasomal ATPases contain tandem repeat of AAA module and are conserved throughout the archeao-eukaryotic branch [49,50]. In the *P. falciparum *genome database, five proteins (PFD0665c, PF10_0081, PF11_0314, PF13_0033 and PF13_0063) have already been identified as Rpt homologues. Another protein PFL2345c, which is annotated as the tat-binding protein homolog, has also been found to be the part of this complex based on its sequence similarity and structural patterns. Secondary structure analysis shows that all six the proteins have classical helical and β-strand pattern (Figure 7). The 'D1 and D2' is another family that contains proteins with two AAA domains named as D1 and D2. This family includes N-ethylmaleimide-sensitive fusion protein (NSF), ATPase family gene (AFG) and Cell Division Cycle 48 (CDC48) proteins [51,52]. The NSF proteins play an important role in vesicle mediated protein trafficking in which the D1 AAA cassette is the active ATPase while D2 is nucleotide binding [47,48]. The only NSF homologue identified in the malaria parasite is PFC0140c that contains the N-domain (essential for soluble NSF-attachment protein binding). The ATPase CDC48 family has two representatives in the *P. falciparum *genome, PF07_0047 and PFF0940c. An additional protein (MAL8P1.92) is observed, having similar sequence features and structural as that of known CDC48 proteins. It shows a significant sequence similarity of MAL8P1.92 with known CDC48 proteins along with the conserved secondary structural features such as the presence of an additional helix downstream of strand-2 in the ATPase domain. Phylogenetic analysis clustered proteins of proteasomal complex in a tight cluster close to another cluster of FtsH proteins (Figure 8).

#### RuvB/TIP49 clade

**Figure 10:**
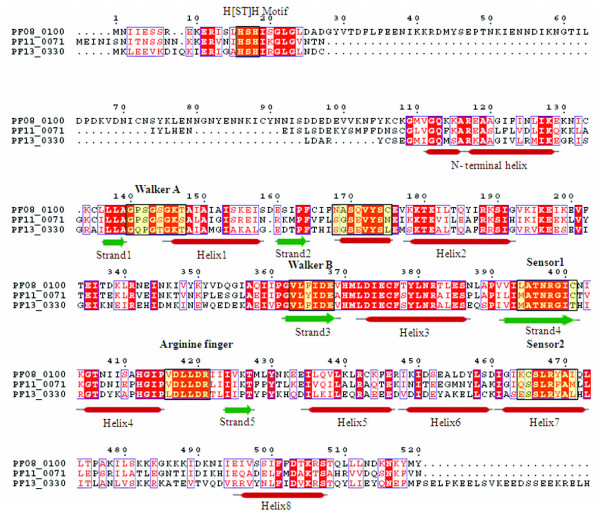
**Multiple sequence alignment of P-loop NTPase domain of *P. falciparum *Tip 49 proteins**. Conserved residues are in solid red and characteristic motifs are boxed in black and shaded in yellow. The corresponding secondary structure is shown below the alignment. Red cylinder represents helices and green arrows represent β-strands. Note the presence of additional helix between strand 2 and helix 2 in these proteins.

The TIP49 ATPases associate with the TATA-binding proteins and appear to play a critical role in the assembly of complexes related to transcriptional activation [53]. Three proteins (PF11_0071, PF08_0100 and PF13_0330) have already been annotated as RuvB DNA helicases in the *P. falciparum *genome. However, presence of certain key features such as conserved 'pre-sensor-1 β hairpin' which is the characteristic of RuvB DNA helicases is not observed in these three proteins. Instead, the N-terminal module with H [ST]H motif, a characteristic of TIP49 family [35,54] is found in these proteins. Furthermore, the multiple sequence alignment based secondary structure analysis showed presence of an additional small helix downstream of strand 2, as in the case of classical AAA clade proteins (Figure 10). These observations suggest that these three proteins should be annotated as TIP49 proteins in the classical AAA clade instead of RuvB DNA helicases.

### MutS proteins/DNA mismatch repair proteins

**Figure 11:**
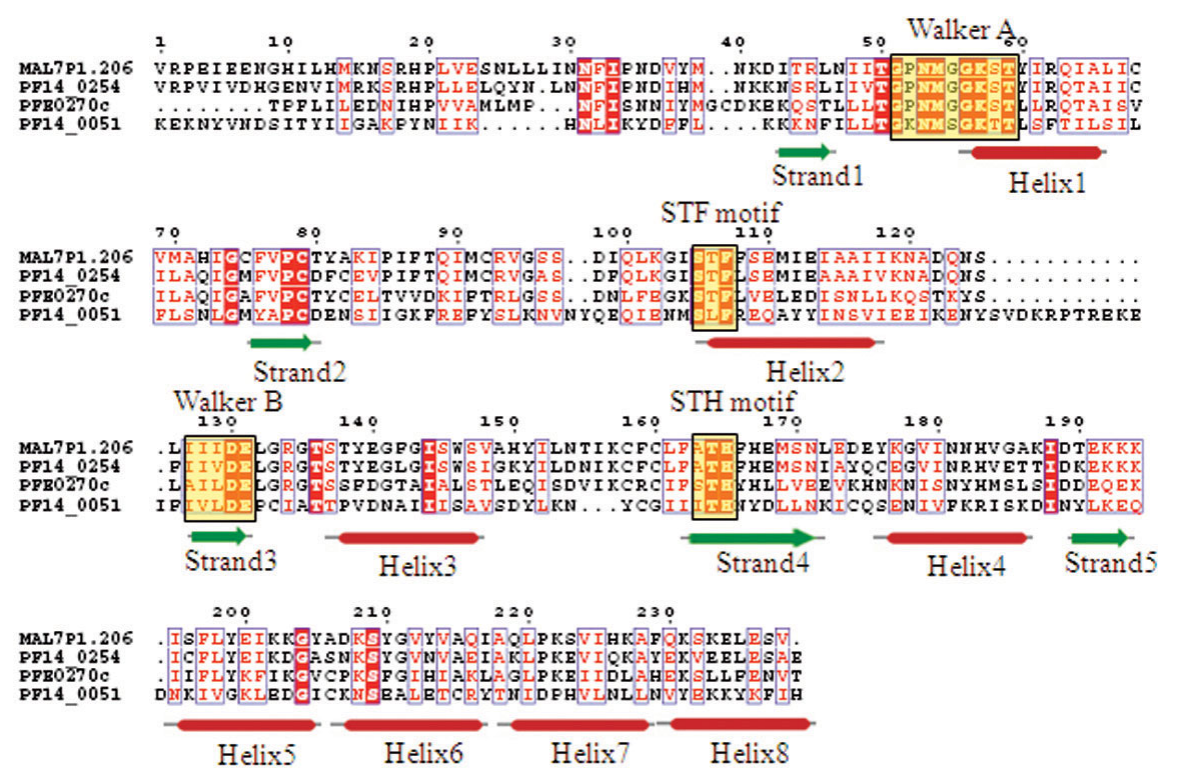
**Multiple sequence alignment of P-loop NTPase domain of *P. falciparum *MutS proteins**. Conserved residues are in solid red and characteristic motifs are boxed in black and shaded in yellow. The corresponding secondary structure is shown below the alignment. Red cylinder represents helices and green arrows represent β-strands.

**Figure 12:**
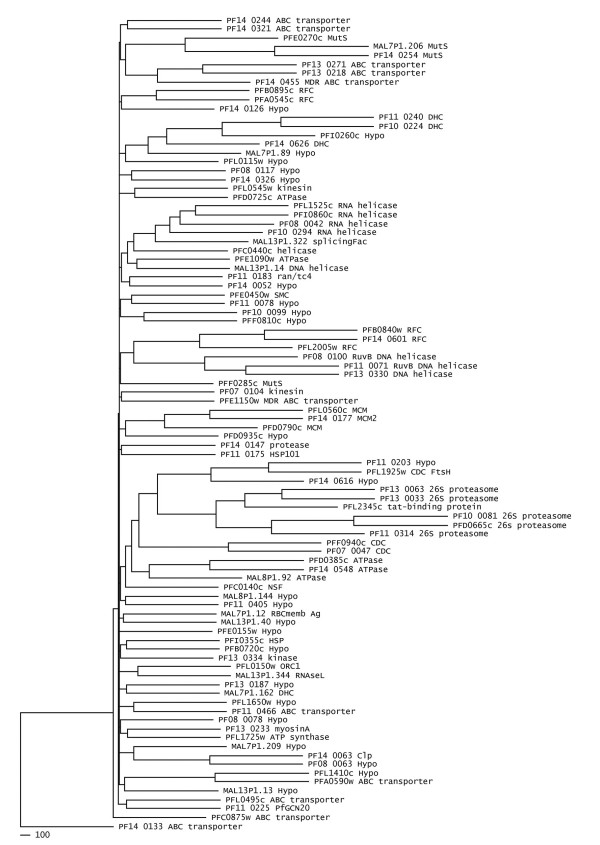
**The dendrogram showing phylogenetic grouping based on the sequence and structural similarity of NTPase domain of *P. falciparum *P-loop NTPases**. The proteins with their respective annotations are mentioned.

The precise cladistic classification of this group of proteins is not known due to the lack of any unique/peculiar feature(s) pertaining to this group of proteins. In the *P. falciparum *genome database, three proteins (MAL7P1.206, PF14_0254 and PFE0270c) have been annotated as DNA mismatch repair proteins. The characteristic motifs (Walker A, STF, Walker B and STH) for this class of proteins are found to be well conserved in all the three proteins. A hypothetical protein PF14_0051 is also found to contain similar domain as other MutS proteins but showed sequence variations all along the length of P-loop domain, including considerable substitution of 'T' → 'L' in the STF motif (Figure 11). Phylogenetic classification based on the secondary structure of the P-loop domains of these proteins grouped them within the cluster of five ABC transporter proteins (Figure 12) and thus together these can be classified under the ABC/SMC superfamily.

### AAA domain sequence and secondary structure based functional classification of hypothetical proteins

**Figure 13:**
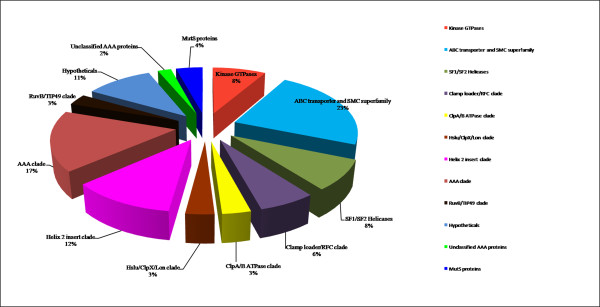
**Functional distribution of P-loop NTPases in the studied dataset**.

The phylogenetic analyses of all the 97 *P. falciparum *P-loop NTPases based upon the secondary structure as well as sequence features of their AAA domain led to an interesting observation. The functional distribution of all 97 proteins is shown as a percentage of total number of proteins for each of the clade studied (Figure 13). Proteins with similar functional roles (or which are parts of larger functional complexes) are found to be grouped together in the analysis. This is remarkably observed for FtsH, ClpA/B and dynein ATPases families. A number of hypothetical proteins with characteristic functional domains are also found to strongly cluster with the annotated proteins of respective families. For example, the two hypothetical proteins having FtsH domains (PF11_0203 and PF14_0616) clustered with the annotated FtsH protein when subjected to both sequence and secondary structure based analyses (Figure 12). A similar result is found for the hypothetical protein PFI0260c, having dynein heavy chain domain, which clusters with four other dynein proteins (Figure 12). These results suggest a correlation between the sequence and structure conservation of the P-loop domain and the functional role of these proteins. Thus it can be speculated that proteins involved in similar functions may group together due to conservation of structural and sequence patterns of their NTPase domains and thus may be suggestive of their putative role. A number of hypothetical proteins that contain only AAA domain are also found to group within clusters of proteins having a defined functional role (Figure 12). These results suggest that the AAA domain sequence and secondary structure homology based phylogenetic grouping may help to infer possible functional role of these AAA proteins and may be used as a complementary method in the annotation process.

Absence of sequence similarity with any other known proteins is a major hurdle in functional classification of large number of *P. falciparum *hypothetical proteins. Martin [55] used hydropathy plots to identify novel membrane transporters from these hypothetical proteins of *P. falciparum*. The possible functional roles for few of *P. falciparum *hypothetical proteins are predicted based upon their grouping with other functionally annotated P-loop proteins in the phylogenetic analysis. The PF14_0126 protein is found to cluster with two functionally annotated RFC proteins and PFD0935c is observed to fall within the cluster of three MCM proteins and thus these proteins might have functional roles as RFC and MCM proteins respectively. Similarly PFF0810c and PF10_0099 are observed to cluster with SMC proteins, MAL13P1.13 with ABC transporters and PF14_0052 with ran/tc4 family proteins, suggesting their possible functional roles as SMC, ABC transporters and ran/tc4 proteins respectively. These hypothetical proteins have shown significant similarities at both the sequence and secondary structural levels. Thus, this approach may be extended to other organisms to classify and assign putative functions to many hypothetical proteins.

### Identification of orthologous protein sequences using OrthoMCL

Firstly, 1,580 different orthologous groups are found between *P. falciparum *and *Homo sapiens*, consisting of 1,683 different proteins from the parasite. Interestingly, 37 *P. falciparum *P-loop NTPases have no ortholog in *H. sapiens*. These proteins include helicases, ABC transporters, Clp protease and heat shock protein. Out of these 37 proteins, 17 are annotated as hypothetical proteins. Together, this analysis offers opportunities to explore the potential of these proteins as novel drug targets without affecting the host.

The OrthoMCL analysis amongst six *Plasmodium *sp. namely *P. falciparum*, *P. vivax*, *P. berghei*, *P. chabaudi*, *P. yoelii *and *P. knowlesi*, identified 88 different *P. falciparum *P-loop NTPase proteins that have at least one ortholog in another malaria parasite species. The remaining nine NTPase proteins of *P. falciparum *are the proteins, which are unique to *P. falciparum *genome. These proteins include ABC transporter, 26S proteasome subunit Rpt3 and DNA replication licensing factor MCM2 in addition to hypothetical proteins. These proteins might be responsible for some dedicated pathways in the life-cycle of the parasite and thus can be of immense interest for further research that may provide clues to a number of unanswered questions in the parasite biology.

Since, there are few characteristic similarities among *P. falciparum *and plants; an attempt is made to identify orthologous of *P. falciparum *P-loop NTPase proteins in *Arabidopsis thaliana *a model organism to compare. A total of 57 parasite NTPase proteins have ortholog in *A. thaliana *genome. Four of these proteins are predicted to be targeted to the apicoplast of the *P. falciparum*. These are cell division cycle ATPase (PF07_0047), heat shock protein (PF11_0175), ATP-dependent transporter (PF14_0133) and a hypothetical protein (PF08_0063). Out of these four proteins, the ATP-dependent transporter protein has no human ortholog. The expression profile of this protein shows the peak expression at late-trophozoite and early schizont stages of the life-cycle. Together, this protein may be considered as a promising drug target.

The orthologous search is further extended to prokaryotes (*Synechococcus sp*., *Mycobacterium tuberculosis*, *Escherichia coli *and *Staphylococcus aureus*) where only 10 *P. falciparum *P-loop NTPases are observed to have orthologs in at least one of these prokaryotes. These 10 NTPases from the parasite showed sequence or structural similarity with bacterial ATPases such as multidrug resistance protein; heat shock protein and cell division protein FtsH protein (present in all eubacterial species). It strongly suggests that few unique processes of the parasite are governed by prokaryotic type mechanisms such as drug-resistance, cell-cycle or protein folding involving heat shock proteins. Some of these prokaryote like-parasite proteins may play crucial role in the parasite life cycle and can be studied as novel drug targets.

### Concluding remarks

P-loop NTPases comprise one of the largest protein families with members present in all kingdoms of life. Numerous subgroups of the family are involved in diverse cellular functions. The functional roles of a number of families belonging to P-loop NTPases superfamily are still unknown in eukaryotes. The repertoire of the P-loop NTPase has not been identified and classified in *P. falciparum*. The challenge of studying and classifying these NTPases further increases in *P. falciparum *due to low sequence similarity and unique features of the genome such as extended insertions and repeats. In the present study, a systematic classification of the P-loop NTPases in *P. falciparum *genome is carried out, that provided information on their function, classification, phylogenetic and orthologous relationships amongst various protein families and organisms. Variations in critical residues within the conserved regions as well as long insertions are observed in the P-loop NTPase domain for most of the *P. falciparum *NTPases suggesting that the parasite has evolved constantly to sustain inspite of the mutations/variations in these imperative regions. The study provided an understanding of the P-loop NTPases, especially in terms of their structural and functional relationships. The proteins with similar functional roles are observed to have similar sequence and structure pattern of P-loop domain. Based on this, putative functional roles for 14 hypothetical proteins are predicted. This is one of the key findings of the study pertaining to the fact that most of *P. falciparum *proteins are not homologous to any other eukaryotic protein and have been annotated as hypothetical proteins. Therefore, elucidation of putative roles of these proteins that are unique to the parasite may provide leads to identify novel drug targets. The sequence orthology based studies are found to be useful in identifying P-loop NTPases either similar to prokaryotic origin or restricted to *Plasmodium *species. Such P-loop NTPases involved in important physiological pathways may lead to identification of new drug targets. It must be emphasized that the current study demonstrates the possible achievements of a computational analysis and is a preliminary investigation. Experimental evidence to explore the role of these genes is thus required. It becomes mandatory in the case of *P. falciparum *where new functional roles have been predicted for a significant number of hypothetical proteins inspite of very low levels of sequence similarity. Overall the study provides us new leads in investigating the functions and biology of *P. falciparum *P-loop NTPases.

## Competing interests

The authors declare that they have no competing interests.

## Authors' contributions

DG^1 ^and MKK carried out the work and drafted the manuscript. DG^2^and VSC critically revised the manuscript content. AM contributed to concept, design and have given final approval of the version to be published.

## Supplementary Material

Additional file 1 **A brief description of different clades of P-loop NTPases showing number of proteins in each class, respective protein ID, full sequence length and the P-loop NTPase region under study**. Additional table. Click here for file

Additional file 2 **Table showing repertoire of P-loop NTPases of *P. falciparum *and their sequence characteristics**. Expression profiles of each protein across the parasitic life cycle based on the transcriptome data as well as their annotation at PlasmoDB are also given. The hypothetical proteins which are assigned functional roles in this study are boxed and highlighted in bold. Proteins orthologous to prokaryotes are marked with ± and those orthologous to *Arabidopsis thaliana *are marked with *. SS, Signal Sequence; TM, trans-membrane region; S, sporozoite; ER, early ring; LR, late ring; ET, early trophozoite; ES, early schizont; LS, late schizont; and M, merozoites. Click here for file

Additional file 3 **Evolutionary classification of *P. falciparum *ClpA/B proteins with seed ClpA/B proteins extracted from Pfam (Pfam domain id- PF10431)**. Additional figure. Click here for file
