# Supplementary material for: A systematic classification of Plasmodium falciparum P-loop NTPases: structural and functional correlation
Source: Malar J. 2009 Apr 18;8:69. doi: 10.1186/1475-2875-8-69 (PMC2674469; doi:10.1186/1475-2875-8-69)
Supplement: Additional file 1 — A brief description of different clades of P-loop NTPases showing number of proteins in each class, respective protein ID, full sequence length and the P-loop NTPase region under study. Additional table. [file 1475-2875-8-69-S1.doc]

| Additional file 1: A brief description of different clades of P-loop NTPases showing number of proteins in each class, respective protein ID, full sequence length and the P-loop NTPase region under study. | | | | | | |
| --- | --- | --- | --- | --- | --- | --- |
|  |  |  |  |  |  |  |
| S.No. | Superfamily/superclade/ clade | Annotated  proteins | Hypothetical  proteins | Protein ID Length  (a.a) | Sequence region studied | General biological functions |
| 1. | Kinase GTPases | 6 | 2 | PFL1435c 2231  PF13_0233 818  PF13_0334 462  PF11_0183 214  PFF0810c 516  PFA0495c 934  PF13_0069 1128  PF14­_0052 575 PF07_0104 1897 | 56-1128  65-818  251-432  5-167  2-478  4-239  196-1041  2-528  240-567 | Cytoskeleton maintenance, initiation and elongation factors and protein kinases |
| 2. | ABC transporter and SMC superfamily | 19 | 3 | PF13­_0218 925  PF13_0271 1049  PFL0495c 855  PFA0590w 1822  MAL13P1.344 619  PFC0875w 3133  PF08_0078 1419  PFE1150w 1419  PF14_0455 1024  PFL1410c 2108  PF11_0225 815  PF14_0321 171  PF11_0466 872  PF14_0133 347  PF14_0244 660  MAL13P1.96 1218  PFD0685c 1193  PFE0450w 1708  PF11_0317 1818  PFF0285c 2236  MAL13P1.13 2743  PF10_0099 1839 | 576-907  811-1048  524-850  1444-1811  339-557  924-1225  969-1267  1126-1402  686-926  1821-2091  597-791  4-171  593-819  100-331  22-247  1-1176  1-1178  63-1655  291-1797  4-2207  1805-2573  691-1291 | Transporters, drug resistance, chromosomal maintenance and repair. |
| 3. | SF1/SF2 Helicases | 8 | 0 | PF10_0294 1290  MAL13P1.322 1151  PF08­_0042 867  PFL1525c 1168  PFI0860c 820  PFC0440c 2269  MAL13P1.14 1566  PF14_0234 1236 | 640-786  488-634  175-617  632-825  317-647  458-527  467-623  9-265 |  |
| 4. | A+ superfamily |  |  |  |  |  |
| 4a | Clamp loader/RFC clade | 6 | 0 | PF14_0601 344  PFL2005w 366  PFB0895c 904  PFA0545c 1167  PFB0840w 330  PFL0150w 1189 | 24-217  27-218  350-602  239-555  15-215  767-1036 | Replication and origin recognition factors. |
| 4b | ClpA/B ATPase clade | 2 | 1 | PF11_0175 906  PF14_0063 1341  PF08_0063 1070 | 579-903  909-1332  757-1067 | Heat shock proteins and Clp proteases. |
| 4c | Pre-sensor-1 β hairpin (PS1BH) superclade |  |  |  |  |  |
|  | Hslu/ClpX/Lon clade | 2 | 1 | PFI0355c 922  PF14_0147 1192  PF14_0126 1219 | 483-922  480-732  418-825 | Heat shock proteins and Clp proteases. |
|  | Helix 2 insert clade | 7 | 4 | PF14_0177 971  PFD0790c 1465  PFL0560c 1024  PFI0260c 6118  PF14_0626 6473  PFL0115w 5729  MAL7P1.162 4971  PF10_0224 5687  PF11_0240 5251  MAL7P1.89 5658 PF14_0326 8094 | 490-868  79-1382  573-927  3593-3859  3241-2800  2421-2634  3037-3391  3777-4077  1895-2081  3068-3280  523-885 | Minichromosomal maintenance proteins and cytoskeletal maintenance proteins. |
| 4d | AAA clade | 14 | 2 | PFD0665c 392  PF10_0081 455  PF11_0314 439  PF13_0033 393  PF13_0063 420  PFL2345c 435  PFF0940c 828  PF07_0047 1229  MAL8P1.92 1224  PFC0140c 783  PF14_0548 419  PF14_0616 706  PFL1925w 880  PF11_0203 1052  PFE0155w 979  PFE1090w 612 | 192-392  194-445  180-431  131-382  149-409  173-430  194-465  928-1195  250-508  244-528  106-401  221-498  128-524  448-846  152-495  62-298 | Proteasomal, metalloproteases, cell division cycle proteins and cellular trafficking proteins. |
|  | RuvB/TIP49 clade | 3 | 0 | PF11_0071 475  PF13_0330 483  PF08_0100 520 | 40-458  37-448  134-327 | DNA helicases. |
| 5. | Hypotheticals without any known function | 10 |  | MAL7P1.209 668  MAL8P1.144 1467  PFD0385c 667  PFL0425c 1753  MAL13P1.40 359  PF08_0117 1203  PF11_0078 1024  PF11_0405 606  PFB0720c 899  PFL1650w 2033 | 324-592  657-984  383-653  1-111  62-141  882-1189  257-826  345-987  319-588  1272-1305 |  |
| 6. | Unclassified A proteins | 2 |  | PFD0725c 379  MAL7P1.12 2283 | 33-366a  1766-2189 |  |
| 7. | MutS proteins | 3 | 1 | MAL7P1.206 873  PF14_0254 811  PFE0270c 1350  PF14_0051 1515 | 572-811  510-748  1083-1313  1013-1256 | DNA repair proteins. |
