# Supplementary material for: A systematic classification of Plasmodium falciparum P-loop NTPases: structural and functional correlation
Source: Malar J. 2009 Apr 18;8:69. doi: 10.1186/1475-2875-8-69 (PMC2674469; doi:10.1186/1475-2875-8-69)
Supplement: Additional file 2 — Table showing repertoire of P-loop NTPases of P. falciparum and their sequence characteristics. Expression profiles of each protein across the parasitic life cycle based on the transcriptome data as well as their annotation at PlasmoDB are also given. The hypothetical proteins which are assigned functional roles in this study are boxed and highlighted in bold. Proteins orthologous to prokaryotes are marked with ± and those orthologous to Arabidopsis thaliana are marked with *. SS, Signal Sequence; TM, trans-membrane region; S, sporozoite; ER, early ring; LR, late ring; ET, early trophozoite; ES, early schizont; LS, late schizont; and M, merozoites. [file 1475-2875-8-69-S2.doc]

**_Additional file 2_:** Table showing repertoire of P-loop NTPases of *P.falciparum* and their sequence characteristics. Expression profiles of each protein across the parasitic life cycle based on the transcriptome data as well as their annotation at PlasmoDB are also given. The hypothetical proteins which were assigned functional roles in this study are boxed and highlighted in bold. Proteins orthologous to prokaryotes are marked with ± and those orthologous to *Arabidopsis thaliana* are marked with *. SS, Signal Sequence; TM, trans-membrane region; S, sporozoite; ER, early ring; LR, late ring; ET, early trophozoite; ES, early schizont; LS, late schizont; and M, merozoites

| **Protein ID** | **SS** | **TM** | **Sub-cellular localization** | **Pfam Domain (e-value)** | **Expression profile** | | | | | | | | **PlasmoDB annotation** |
| --- | --- | --- | --- | --- | --- | --- | --- | --- | --- | --- | --- | --- | --- |
|  |  |  |  |  | S | ER | LR | ET | LT | ES | LS | M |  |
| **Kinase GTPases** | | | | | | | | | | | | | |
| PFL1435c | - | - |  | Myosin head (4.9e-17) | + | - | - | - | - | + | +++ | ++ | myosin d |
| PF13-0233 | - | - |  | Myosin head  (2.0e-178) | ++ | - | - | - | - | - | ++ | ++ | myosin a |
| PF13-0334* | - | - |  | PNK3P (8.10e-74) | - | + | + | + | ++ | - | - | - | polynucleotide kinase, putative |
| PF11-0183* | - | - | Nuclear | Ras (1.9e-64) | + | + | + | + | ++ | ++ | + | + | GTP-binding nuclear protein ran/tc4 |
| **PFF0810c** | **-** | **-** |  | **Ras(2.00e-05)** | **-** | **-** | **-** | **-** | **-** | **-** | **-** | **-** | **hypothetical protein** |
| PFA0495c | - | - |  | GTP-EFTU | ++ | - | +++ | - | +++ | ++ | - | - | selenocysteine-specific elongation factor selB homologue, putative |
| PF13-0069 | - | - | Mitochondria | GTP-EFTU  (9.9e-16) | - | - | - | - | - | - | - | - | translation initiation factor if-2, putative |
| **PF14-0052*±** | **-** | **-** |  | **CobW (2e-13)** | + | ++ | +++ | ++ | ++ | + | + | + | **hypothetical protein, conserved** |
| PF07-0104 | - | - |  | AAA (5.20e-90) | - | - | - | - | - | - | - | - | kinesin-like protein, putative |
| **ABC transporters/SMC family** | | | | | | | | | | | | | |
| PF13-0218 | - | **Y** |  | ABC transporter | - | - | - | - | - | - | - | - | ABC transporter, putative |
| PF13-0271 | - | **Y** | Mitochondria | MobB (0.029) | - | - | - | - | ++ | - | +++ | - | ABC transporter, putative |
| PFL0495c* | - | **Y** |  | MobB (0.0041) | + | - | + | ++ | +++ | +++ | ++ | + | ABC transporter, putative |
| PFA0590w | - | **Y** | Mitochondria | PRK (0.0055) | - | - | - | + | ++ | +++ | ++ | - | ABC transporter, putative |
| MAL13P1.344* | - | - | Mitochondria | RL1(5.5e-12) | + | + | ++ | ++ | +++ | ++ | + | + | RNAse L inhibitor protein, putative |
| PFC0875w | **-** | **Y** | Mitochondria | NACHT (0.0027) | + | + | ++ | +++ | ++ | - | - | ++ | transporter, putative |
| PF08-0078 | **Y** | **Y** | Apicoplast | NACHT (0.015) | + | + | + | ++ | +++ | +++ | + | + | hypothetical protein |
| PFE1150w*± | - | **Y** |  | MMR-HSR1 (0.0016) | - | + | + | + | + | + | + | +++ | multidrug resistance protein |
| PF14-0455* | - | **Y** |  | ABC transporter | + | - | + | ++ | +++ | + | - | - | multidrug resistance protein 2 |
| **PFL1410c** | **-** | **Y** | Mitochondria | **Miro (0.0084)** | **+** | **+** | **++** | **+** | **+** | **+** | **+** | **++** | **hypothetical protein** |
| PF11-0225*± | - | - |  | Miro(0.00079) | + | + | + | ++ | + | + | + | ++ | PfGCN20 |
| PF14-0321* | - | - |  | Miro(0.15) | + | - | + | ++ | ++ | +++ | + | + | ABC transporter, putative |
| PF11-0466 | **Y** | **Y** |  | ABC transporter | - | - | - | - | +++ | - | - | - | transport protein, putative |
| PF14-0133*± | - | **Y** |  | ABC trans  (8.00e-26) | + | - | - | - | + | +++ | + | - | ATP-dependent transporter, putative |
| PF14-0244* | - | **Y** |  | ABC-tran  (3.30e-40) | + | - | - | - | + | + | - | - | ABC transporter, putative |
| MAL13P1.96* | - | - |  | SMC-N (3.7e-98) | + | - | - | + | ++ | +++ | ++ | + | chromosome segregation protein, putative |
| PFD0685c* | - | - | Mitochondria | SMC M (1.1e-50) | + | - | - | - | ++ | +++ | + | + | chromosome associated protein, putative |
| PFE0450w* | - | - |  | SMC-N (1.3e-101) | + | - | - | - | ++ | ++ | + | - | chromosome condensation protein, putative |
| PF11-0317* | - | - |  | SMC-N (1.1e-64) | + | - | + | + | ++ | ++ | + | + | structural maintenance of chromosome protein, putative |
| PFF0285c* | - | - |  | SMC-N | - | - | - | - | - | - | - | - | DNA repair protein RAD50 |
| **SF1/SF2 Helicases** | | | | | | | | | | | | | |
| PF10-0294* | - | - |  | Helicase RNA  (3.2e-28) | + | + | + | + | ++ | + | + | + | RNA helicase, putative |
| MAL13P1.322 * | - | - |  | HA2 (2.6e-26) | +++ | ++ | + | ++ | ++ | + | + | ++ | Splicing factor, putative |
| PF08-0042 | - | - | Mitochondria | HA2 (1.9e-22) | + | - | ++ | - | - | - | - | + | ATP-dependent RNA helicase prh1, putative |
| PFL1525c | - | - |  | HA2 (3.9e-24) | + | - | ++ | - | ++ | ++ | ++ | + | pre-mRNA splicing factor RNA helicase, putative |
| PFI0860c* | - | - |  | HA2 (9.60e-27) | - | ++ | ++ | ++ | +++ | - | - | - | ATP-dependant RNA helicase, putative |
| PFC0440c | - | - |  | HA2 (6.20e-20) | - | + | ++ | ++ | +++ | +++ | + | - | helicase, putative |
| MAL13P1.14* | - | - |  | Helicase C  (1.3e-13) | + | ++ | ++ | +++ | +++ | - | - | ++ | ATP-dependent DEAD box helicase, putative |
| PF14-0234 | - | - |  | DEAD box | + | + | + | + | ++ | ++ | +++ | ++ | DNA-directed DNA polymerase, putative |
| **AAA+ superfamily**  Clamp loader/RFC clade | | | | | | | | | | | | | |
| PF14-0601*± | - | - |  | Rep-fac-C  (2.3e-30) | + | - | - | + | +++ | ++ | + | - | replication factor C3 |
| PFL2005w* | - | - |  | Rep FactorC (3.7e-16) | - | - | - | +++ | ++ | + | - | - | replication factor c subunit 4 |
| PFB0895c* | - | - |  | RFC (1.60e-72) | + | - | - | - | ++ | +++ | + | - | replication factor C subunit 1, putative |
| PFA0545c* | - | **-** |  | NACHT (0.0092) | - | - | - | - | ++ | + | + | - | replication factor c protein, putative |
| PFB0840w* | - | - |  | Rep FactorC (2.5e-23) | - | - | - | - | ++ | +++ | + | - | replication factor C, subunit 2 |
| PFL0150w* | - | - |  | DNA Pol B (0.032) | + | - | - | + | +++ | +++ | ++ | + | origin recognition complex 1 protein |
| ClpA/B ATPase clade | | | | | | | | | | | | | |
| PF11-0175*± | **Y** | **Y** | Apicoplast | Clp N (3.1e-05) | + | + | ++ | ++ | + | - | + | ++ | heat shock protein 101, putative |
| PF14-0063 | **-** | **Y** | Apicoplast | Clp-N (0.0056) | + | - | + | + | +++ | +++ | ++ | + | ATP-dependent Clp protease, putative |
| **PF08-0063*±** | **Y** | **Y** | **Apicoplast** | **Clp-N (0.00012)** | **-** | **-** | **+** | **+** | **++** | **+** | **+** | **+** | **hypothetical** |
| **Pre-sensor 1 β-hairpin superclade** | | | | | | | | | | | | | |
| *Hslu/ClpX/Lon* clade | | | | | | | | | | | | | |
| PFI0355c | - | - |  | Mg-chelatase (0.011) | - | - | ++ | - | - | - | ++ | - | ATP-dependent heat shock protein, putative |
| PF14-0147* | - | - | Mitochondria | AAA (6.80e-17) | + | - | - | ++ | ++ | ++ | + | - | ATP-dependent protease, putative |
| **PF14-0126** | **-** | **-** |  | **AAA (2.6e-76)** | **+** | **-** | **+** | **+** | **++** | **+** | **-** | **-** | **hypothetical protein, conserved** |
| **Helix 2 insert clade** | | | | | | | | | | | | | |
| PF14-0177* | - | - |  | MCM (1.3e-173) | + | - | - | + | ++ | +++ | ++ | + | DNA replication licensing factor MCM2 |
| PFD0790c | - | - |  | MCM (6.70e-59) | - | - | - | - | ++ | +++ | - | - | DNA replication licensing factor, putative |
| PFL0560c | **Y** | - |  | MCM (6.60e-87) | +++ | - | - | - | ++ | ++ | - | - | minichromosome maintenance protein, putative |
| **PFI0260c** | **-** | **-** |  | **DHC-N2 (2.5e-77)** | **-** | **-** | **-** | **-** | **-** | **-** | **-** | **-** | **hypothetical protein** |
| PF14-0626 | - | - |  | DHC (1.5e-127) | - | - | - | - | - | - | - | - | dynein beta chain, putative |
| **PFL0115w** | **-** | **-** |  | **DHC-N2 (2.6e-79)** | **-** | **-** | **-** | **-** | **-** | **-** | **-** | **-** | **hypothetical protein** |
| MAL7P1.162 | - | - |  | DHC-N1  (3.5e-200) | ++ | - | - | - | - | - | - | - | dynein heavy chain, putative |
| PF10-0224 | - | - |  | Dynein heavy  (1.10e-241) | - | - | - | - | - | - | - | - | dynein heavy chain, putative |
| PF11-0240 | - | - |  | Dynein heavy  (1.40e-93) | ++ | - | + | - | ++ | +++ | - | + | dynein heavy chain, putative |
| **MAL7P1.89** | **-** | **Y** |  | **DHC-N2**  **(7.90e-119)** | **-** | **-** | **-** | **-** | **-** | **-** | **-** | **-** | **hypothetical protein, conserved** |
| PF14-0326* | - | **Y** |  | AAA-3 (7.1e-07) | - | - | - | +++ | - | - | +++ | - | hypothetical protein |
| **AAA clade** | | | | | | | | | | | | | |
| PFD0665c* | - | - |  | AAA (3.90e-87) | ++ | + | ++ | + | ++ | +++ | ++ | + | 26s proteasome aaa-ATPase subunit Rpt3, putative |
| PF10-0081* | - | - |  | AAA (1.30e-87) | +++ | + | + | + | ++ | +++ | ++ | + | 26S proteasome regulatory subunit 4, putative |
| PF11-0314* | - | - |  | Mg-chelatase (0.0003) | + | + | + | ++ | +++ | +++ | +++ | + | 26S protease subunit regulatory subunit 6a, putative |
| PF13-0033* | - | - | Mitochondria | Mg-chelatase  (6.7e-05) | + | + | + | + | ++ | +++ | ++ | + | 26S proteasome regulatory subunit, putative |
| PF13-0063* | - | - |  | Mg-chelatase (0.0046) | + | + | + | + | ++ | +++ | ++ | + | 26S proteasome regulatory subunit 7, putative |
| PFL2345c* | - | - |  | Mg-chelatase (0.0046) | - | + | + | + | ++ | +++ | +++ | + | tat-binding protein homolog |
| PFF0940c* | - | - |  | CDC48-N  (1.3e-31) | - | - | - | - | - | - | - | - | cell division cycle protein 48 homologue, putative |
| PF07-0047* | **Y** | **Y** | Apicoplast | Mg-chelatase  (3.8e-05) | + | - | + | ++ | +++ | ++ | + | + | CDC ATPase |
| MAL8P1.92* | - | **Y** |  | ParvoNS1 (0.0002) | + | + | ++ | ++ | +++ | + | - | ++ | ATPase,putative |
| PFC0140c* | - | - |  | Mg-chelatase (0.00068) | + | + | + | + | ++ | +++ | + | + | N-ethylmaleimide-sensitive fusion protein, putative |
| PF14-0548* | - | - |  | MIT (2.4e-09) | ++ | - | - | + | + | + | ++ | + | ATPase, putative |
| **PF14-0616*±** | **-** | **-** |  | **Peptidase-M41**  **(4.1e-94)** | + | + | ++ | +++ | + | + | ++ | + | **Hypothetical protein** |
| PFL1925w*± | - | **Y** |  | Peptidase-M41  (4.1e-94) | + | - | - | ++ | ++ | +++ | - | + | cell division protein FtsH, putative |
| **PF11-0203*±** | **-** | **Y** |  | **Peptidase-M41**  **(2.9e-80)** | **+** | **-** | **+** | **+** | **++** | **+** | **+** | **-** | **hypothetical protein** |
| PFE0155w | - | - |  | Calpain-II (0.0063) | + | - | - | - | + | - | + | - | hypothetical protein |
| PFE1090w* | - | - |  | AFG1-ATPase  (9.7e-14) | - | - | - | - | - | - | - | - | nucleotide binding protein, putative |
| *RuvB/TIP49* clade | | | | | | | | | | | | | |
| PF11-0071* | - | - |  | TIP49 (2.7e-220) | + | + | + | ++ | +++ | + | + | + | RuvB DNA helicase, putative |
| PF13-0330* | - | - |  | RuvB (2.1e-223) | - | + | + | + | ++ | - | - | - | ATP-dependent DNA helicase, putative |
| PF08-0100 | - | - |  | TIP49 (3.30e-210) | + | - | - | - | - | - | - | - | ruvB-like DNA helicase, putative |
| **Hypothetical proteins with AAA domains** | | | | | | | | | | | | | |
| MAL7P1.209* | - | - |  | AAA (1.60e-26) | - | - | - | - | - | - | - | - | hypothetical |
| MAL8P1.144 | - | **Y** |  | NACHT (.00067) | - | - | - | + | + | + | ++ | +++ | hypothetical |
| PFD0385c* | - | - |  | Mg-chelatase (0.00059) | - | - | - | - | - | - | - | - | AAA family ATPase, putative |
| PFL0425c | - | - |  | AAA-5 (0.51) | - | - | - | - | - | - | - | - | hypothetical protein |
| MAL13P1.40* | - | - |  | AAA (0.16) | - | - | - | - | - | - | - | - | hypothetical protein |
| PF08-0117 | - | - |  | AAA (2.50e-10) | ++ | + | ++ | - | +++ | - | - | - | hypothetical protein |
| PFL1650w | - | - | Mitochondria | ERF (0.0053) | ++ | - | - | - | + | ++ | + | - | hypothetical protein |
| PF11-0078 | - | - |  | AAA (0.00019) | - | - | - | - | - | - | - | - | hypothetical protein |
| PF11-0405* | - | - |  | AAA (2.00e-26) | ++ | - | - | - | - | - | - | - | hypothetical protein, conserved |
| PFB0720c | - | - |  | AAA-2 (0.37) | + | - | - | - | ++ | + | + | - | hypothetical protein, conserved |
| PFD0725c* | - | - |  | ArsA-ATPase  (4.60e-100) | - | - | ++ | - | - | - | - | - | arsenical pump-driving ATPase, putative |
| MAL7P1.12* | - | **Y** |  | AAA (0.22) | + | + | ++ | +++ | ++ | ++ | + | + | erythrocyte membrane-associated antigen |
| **MutS proteins** | | | | | | | | | | | | | |
| MAL7P1.206* | - | - |  | MutS-V (1.5e-131) | - | - | - | - | - | - | - | - | DNA mismatch repair enzyme, putative |
| PF14-0254* | - | - |  | MutS-V (7.7e-125) | + | - | - | + | +++ | +++ | + | - | DNA mismatch repair protein Msh2p, putative |
| PFE0270c* | - | - |  | MutS-V (6.6e-102) | + | - | + | + | +++ | +++ | + | + | DNA repair protein, putative |
| **PF14-0051** | **Y** | **-** |  | **MUT S-I (1.3e-06)** | **-** | **-** | **-** | **-** | **+++** | **-** | **-** | **-** | **hypothetical protein, conserved** |
| **MAL13P1.13** | **-** | **-** |  | **AAA-5 (0.025)** | **-** | **+** | **+** | **+** | **++** | **++** | **+** | **-** | **hypothetical** |
| **PF10-0099** | **-** | **-** |  | **AAA (0.00015)** | **-** | **-** | **-** | **+** | **-** | **++** | **-** | **-** | **hypothetical** |

­­­­­­­
