# Supplementary figures and images for: A systematic classification of Plasmodium falciparum P-loop NTPases: structural and functional correlation
Source: Malar J. 2009 Apr 18;8:69. doi: 10.1186/1475-2875-8-69 (PMC2674469; doi:10.1186/1475-2875-8-69)

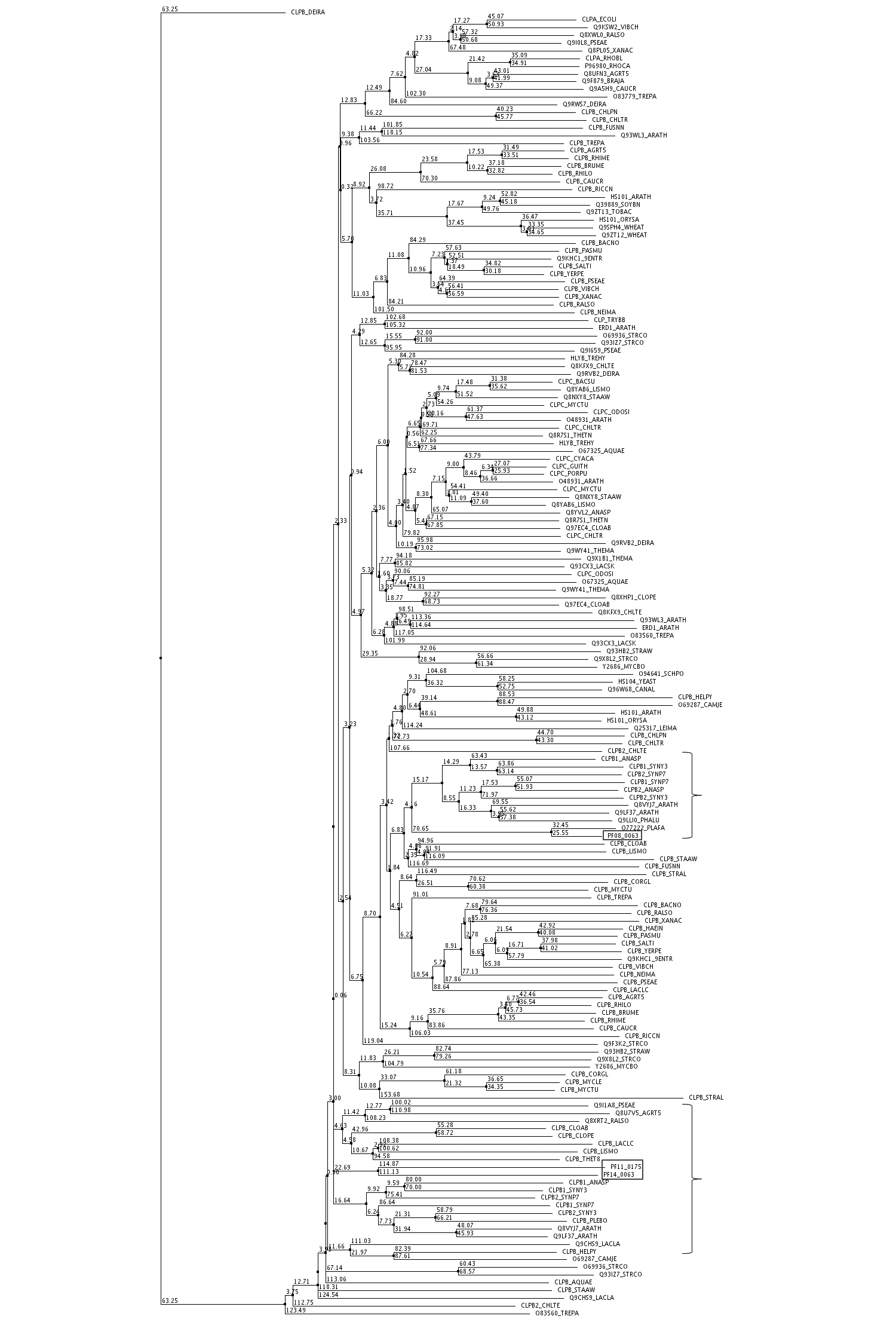

Supplement: Additional file 3 — Evolutionary classification of P. falciparum ClpA/B proteins with seed ClpA/B proteins extracted from Pfam (Pfam domain id- PF10431). Additional figure. [file 1475-2875-8-69-S3.tiff]
